# The role of health literacy in the association between nationality and health status among university students: a cross-sectional study

**DOI:** 10.1080/07853890.2026.2643976

**Published:** 2026-03-26

**Authors:** Akindele Abimibayo Adeoya, Haruki Momma, Ryoichi Nagatomi, Yosuke Yamada

**Affiliations:** ^a^ Department of Medicine and Science in Sports and Exercise, Graduate School of Medicine, Tohoku University, Sendai, Miyagi, Japan; ^b^ Division for Interdisciplinary Advanced Research and Education, Tohoku University Advanced Graduate School, Sendai, Miyagi, Japan; ^c^ Division of Biomedical Engineering for Health and Welfare, Tohoku University Graduate School of Biomedical Engineering, Sendai, Miyagi, Japan; ^d^ Department of Sports and Health Sciences, Graduate School of Biomedical Engineering, Tohoku University, Sendai, Miyagi, Japan

**Keywords:** Health status, nationality, health literacy, international students, Japanese students, university students

## Abstract

**Objective:**

Immigrants often face numerous health-related challenges, which may result in a lower health status (HS) as compared with the domestic population. This study examined the association between nationality and HS among university students in Japan and the role of health literacy (HL) on this association.

**Methods:**

Web-based self-administered surveys were conducted with 1,366 university students across six of Japan’s eight regions between February 20 and August 10, 2023. Nationality and HS were measured using a self-reported questionnaire, and HS was categorized into two groups: good or not good. HL was assessed by the 47-item European Health Literacy Survey.

**Results:**

International students (61%) had a lower prevalence of good HS than Japanese students (73%) (*p* < 0.0001). Even after adjusting for sociodemographic and educational variables, international students showed a lower prevalence of good HS as compared with Japanese students (odds ratios [95% confidence interval]: 0.72 [0.53, 0.99], *p* = 0.04). However, when considering HL level, the association attenuated (*p* = 0.2), and the interaction of HL and nationality was observed (*p* < 0.0001). A subgroup analysis showed that international students had a higher prevalence of good HS as compared to Japanese students among the students with sufficient HL level (1.63, [1.03, 2.58], *p* = 0.03), whereas among the students with inadequate HL level, international students had a lower prevalence of good HS (0.34, [0.20, 0.58], *p* < 0.0001).

**Conclusion:**

Although international students had a lower prevalence of good HS as compared to Japanese students, the direction of the association differs based on the level of HL.

## Introduction

Immigrants often face numerous health-related challenges. For instance, they are less likely to utilize healthcare services, including preventive care such as regular dental checkups [1,2] and cancer screenings [3], compared to the domestic population. Furthermore, studies have shown that foreign nationals have a higher prevalence of hepatitis A and B [4], diabetes, hyperlipidemia, obesity, coronary heart disease and heart failure [5], and poorer periodontal health [6] depending on the country of residence. They are also prone to mental health issues, including frequent exposure to potentially traumatic events, post-traumatic stress disorder, anxiety, depression, somatization, and post-migration living difficulties [7,8]. Given this, foreign nationals will likely face health-related burdens, which can result in lower well-being.

Health status (HS) is a critical measure of overall well-being, commonly assessed using subjective measures such as self-reported questionnaires [9–12]. Yamada and colleagues found a strong association between self-reported HS and the prevalence of lifestyle-related diseases and abnormalities, including cancer, cardiovascular disease, obesity, blood pressure, and glucose metabolism [13]. Furthermore, HS levels predict healthcare needs and utilization [12,14,15], psychological health [11,12,15], morbidity [9,12, 15,16], and mortality [9,10, 17–19]. Young adults are generally unlikely to have lifestyle-related diseases or serious health conditions. However, HS, especially subjective HS, is important for this group as it may directly influence their motivation, vitality, and activities in daily life.

Health literacy (HL) encompasses a broad set of skills and competencies that enable individuals to access, understand, evaluate, and apply health information effectively to make informed decisions concerning healthcare, disease prevention, and health promotion, in order to maintain or improve their health during the life course [20]. While literacy skills enhance an individual’s ability to access, understand, and act upon new information, these skills alone do not guarantee consistent application in situations that require specific content knowledge or in unfamiliar settings [21]. It is recognized as a multidimensional construct that integrates cognitive, social, and behavioral skills, and include interactive and essential competencies needed for navigating complex health systems and play a functional role in health promotion [20–22].

HS is influenced by several factors such as lifestyle [23] and HL [24,25]. Most students do not follow a healthy lifestyle [26]; university students often experience increased stress, unhealthy dietary habits, lack of physical activity, poor sleep patterns, and behavioral risk factors for cardiovascular disease [27,28]. Moreover, the level of HL among students differs based on sociodemographic and economic characteristics [22,29], and academic achievements [30]. Especially, international students are likely to report lower HS than domestic students, because they leave familiar environments and confront issues such as language barriers, different living habits, weather variations, difficulties in accepting and trying new things, rebuilding social capital, and overcoming interpersonal communication challenges. Considering these factors, HS may vary among students depending on their characteristics, lifestyle, and background.

Based on this assumption that HS may vary among students, it is hypothesized that international students would exhibit lower HS. However, a previous study found that international students reported a lower proportion of poor HS compared to domestic students [31], which was inconsistent with the hypothesis. In addition, a more recent study, by Umami and colleagues [32] found no difference in HS between international and domestic medical students. This finding is also contrary to the hypothesis. However, no conclusion can be drawn, because the geographical representativeness was limited to a single university population. In addition, these studies did not consider covariates or confounding factors. In particular, HL may be an important factor for these students because the HL level was associated with both HS [24,25], and nationality [22,33]. HL level may have an effect modification on the association between nationality and HS among university students.

Therefore, the objectives of this study were (1) to investigate the association between nationality and HS, and (2) to examine the effect modification of HL level on the association between nationality and HS among university students in Japan.

## Materials and methods

### Study design and participants

This cross-sectional study included Japanese and international students in Japanese universities. It forms part of the J-IMPACT study (Japan International Migrant Population and Comparative Health Study) [22]. The data were collected from February 20 to August 10, 2023. The first page of the survey link contained information on the purpose, data confidentiality, and ethical requirements of the study. University students willing to complete the survey indicated their informed consent by pressing the consent button before accessing the questionnaire. All instruments were available in both English and Japanese versions.

### Sampling procedure

The study adopted a multi-stage sampling approach. First, the 30 universities with the largest international student populations were identified using Japan Student Services Organization (JASSO) data [34]. Seven institutions were randomly selected to ensure regional diversity across six of Japan’s eight regions (Kanto, Kinki, Kyushu, Tohoku, Hokkaido, and Chubu). These institutions are public national universities with broadly comparable administrative structures and international student compositions. The eligibility criterion was being a currently enrolled student in Japanese universities. At each university, to ensure that only eligible students participated and to minimize duplicate responses, the online questionnaire was self-administered by the researcher and/or research assistants on campus using convenience sampling and accessed *via* QR codes. In total, 1,366 students completed the survey. Because recruitment relied on convenience sampling, the number of students approached could not be determined; therefore, a response rate cannot be estimated.

### Instruments

#### Health status (HS)

Participants were asked to self-report their HS on a 5-point scale: excellent, good, moderate, poor, or very poor. Self-reported HS is a widely utilized measure of general health in both the general population [35] including immigrants [36] and among university students [24]. Previous studies have confirmed that self-reported HS is valid [37], reliable [38], and consistent with objective HS among the general population [39,40]. Participants were categorized into groups of ‘good’ (excellent, good) and ‘not good’ (moderate, poor, very poor) HS. This approach enhances statistical robustness, facilitating comparison across studies, and aligning with epidemiological practices. Dichotomizing self-rated HS categories is an effective strategy for increasing reliability [41]. Indeed, a systematic review found that dichotomized self-rated HS has demonstrated stronger predictive validity for mortality compared to multi-category formats [19]. Nonetheless, to assess the robustness of findings and address potential information loss from dichotomization, HS was additionally analyzed using the original five response categories (coded 0–4) as an ordinal measure, in sensitivity analyses.

##### Nationality

Nationality was obtained *via* a self-reported questionnaire. Participants were asked to choose between two options to indicate their status: Nationality (Japanese or International student).

##### Health literacy (HL)

HL was assessed using the 47-item European Health Literacy Survey Questionnaire (HLS-EU-Q47) [42]. This tool has been extensively validated and used among both general populations [43] and university students [22,25] in Japan. In the present dataset, the internal consistency of the instrument was excellent, with Cronbach’s alpha values of .94 for the English, Japanese, and combined language versions [22]. Items were scored on a 4-point Likert scale ranging from “very difficult” to “very easy”. The mean score varied from 1 to 4 because the lowest possible mean score was 1 and the highest possible mean score was 4. Using scores from all 47 questionnaire items (e.g. finding information about symptoms of illnesses that concern you; understanding health warnings about behaviors such as smoking, low physical activity, and drinking too much; and judging whether your lifestyle affects your health and well-being), a comprehensive general index of HL was constructed. The index score was standardized to a unified metric ranging from 0 to 50 using the following formula: (MEAN–1) × (50/3) [44]. Responses of “do not know” were treated as missing values similar to previous studies [22,43]. HL index scores were calculated only for respondents who provided valid answers to at least 80% of the items associated with all indices for general HL (GEN-HL) (*n* = 1307). Although previous studies generally categorized HL into four groups: inadequate (0–25), problematic (>25–33), sufficient (>33–42), and excellent (>42–50) [22,42,43], we merged the problematic, sufficient, and excellent categories into one due to the small proportions of individuals in the sufficient and excellent categories (7% and 1%, respectively) [22]. In addition, the median score of GEN-HL in our study was 23.1. Thus, we categorized HL into two groups: inadequate (0–25) and sufficient (>25–50). In sensitivity analyses, HL was also modeled as a continuous variable using the total HL score to examine whether observed associations were robust to alternative operationalizations.

##### Sociodemographic characteristics

Sociodemographic characteristics included gender (male, female, others) and age categories (<20 years, 20–24 years, 25–30 years, and ≥31 years). These age ranges were selected based on the average age of first-time students entering higher education, typically 18 years old, and the typical age range for tertiary education attainment of 25–34 years, as reported by the Organization for Economic Co-operation and Development [45]. For the analysis, the university location by prefecture (Tokyo, Osaka, Fukuoka, Ibaraki, Miyagi, Hokkaido, and Aichi) was dichotomized into megacities (Tokyo and Osaka) and metropolitan cities (Fukuoka, Ibaraki, Miyagi, Hokkaido, and Aichi); the program of study was dichotomized into medical sciences (including medicine, biomedical sciences, nursing, pharmacy, medical technology, public health, other allied health sciences) and non-medical (including engineering sciences, ICT, linguistic and cultural studies, communication arts, mathematics, natural sciences, law, economics, criminology, public administration, political science, social sciences, psychology, education, accountancy, business management, etc.); study level was dichotomized into undergraduate and postgraduate; and marital status was categorized as single or married. Religion was dichotomized into adherents (Christianity, Islam, Hinduism, Buddhism, Other) and non-adherents. Parents’ highest level of education was categorized as no education/compulsory education (elementary/junior high school), high school, junior college/vocational school, or university degree or higher. Self-rated economic status was ranked on a 10-point scale ranging from 1 (lowest) to 10 (highest). The results were categorized as low (1–4 points), moderate (5–7 points), or high (8–10 points). Length of stay in Japan was categorized as ≤1 year, 1–2 years, 3–4 years, or 5 years or more. This categorization was chosen because the majority (79%) of international university students in the target population in Japan are enrolled in postgraduate programs [34], which typically last up to five years. Japanese language ability was self-reported and categorized into five levels: 1: cannot speak Japanese, 2: can speak it a little, 3: able to communicate about daily life, 4: able to report or understand information using medical terms, and 5: speaks it fluently [22].

### Data analysis

All data were analyzed using JMP version 17.2.0 (Statistical Discovery LLC, Cary, NC, USA), employing both descriptive and inferential statistical methods. Data were presented as N (%) for categorical variables. The Chi-squared test was used to compare the characteristics of international and Japanese students and to examine the association between sociodemographic variables and HS. T-tests were employed to compare HL indices between Japanese and international students. A multiple logistic regression analysis was performed to examine the association between nationality and HS. First, we considered age and sex (Model 1). Next, we included sociodemographic variables, such as economic status, marital status, level of study, and program of study (Model 2). In Model 3, HL was additionally considered. Interaction effects were assessed between nationality and HL in the fully adjusted model, and adjusted predicted probabilities of good HS were estimated from this model. For model diagnostics, multicollinearity was assessed using correlation matrix diagnostics, with correlation coefficients < 0.70 considered indicative of no problematic collinearity. Model discrimination was evaluated using the area under the receiver operating characteristic curve (AUC). Calibration was assessed using the Hosmer–Lemeshow goodness-of-fit test. Influential observations were examined using Cook’s distance and standardized residuals. Sensitivity analyses excluding influential cases were conducted to assess the robustness of model estimates. In addition, a subgroup analysis was performed based on the level of HL. Similarly, for the subgroup analysis, age and sex were considered (Model 1). In Model 2, additional sociodemographic variables, such as economic status, marital status, level of study, and program of study were considered . Model fit was examined using likelihood-based statistics, including the likelihood ratio test, −2 log likelihood, and information criteria (AICc and BIC). Pseudo-R^2^ statistics were examined to assess overall explanatory power. Diagnostic indices indicated acceptable model fit across all models. Sensitivity analyses were conducted to assess the robustness of findings to alternative operationalizations of HS and HL. HS was re-specified as an ordinal outcome and HL was re-specified as a continuous predictor. All sensitivity models retained the same covariate structure and analytical strategy as the primary analyses. The variables used for these models were selected based on their use in previous studies on HS [9,10,12,31], and HL [22,29], as well as their potential influence on the association in the current study. Statistical significance was set at *p* < 0.05.

### Ethical consideration

The study was conducted in full compliance with the principles of voluntariness, confidentiality, and respect for human subjects, ensuring the protection of the respondents’ legitimate rights and interests. Before the commencement of the study, approval was obtained from the Tohoku University Research and Ethics Committee (approval number: 2022-1-921).

## Results

### Sociodemographic characteristics of participants by nationality

The participants’ sociodemographic characteristics by nationality are shown in Table 1. The study comprised 1366 university students in Japan, including 859 (63%) Japanese students and 507 (37%) international students. The proportion of female, postgraduate, non-medical, married, and religious adherents was higher among international students than among Japanese students (*p* < 0.001). Conversely, international students had lower economic status and parental education levels than Japanese students (*p* < 0.001). No differences were found by city type.

**Table 1.** Sociodemographic characteristics of participants by nationality.

Variable	Total		Japanese		International		Pa
	*N* = 1366	100%	*n* = 859	63%	*n* = 507	37%	
Gender							0.001^a^
Male	732	53.0	497	58.0	235	46.4	
Female	612	45.0	347	40.0	265	52.3	
Others	22	2.0	15	2.0	7	1.3	
Age-group (years)							0.001^a^
< 20	305	22.0	288	34.0	17	3.3	
20–24	706	52.0	511	59.0	195	38.4	
25–30	237	17.0	47	5.0	190	37.4	
> =31	118	9.0	13	2.0	105	21.0	
Level of study							0.001^a^
Undergraduate	881	64.0	717	83.0	164	32.0	
Postgraduate	485	36.0	142	17.0	343	68.0	
Program of study							0.001^a^
Medical	291	21.0	238	28.0	53	10.0	
Non-medical	1075	79.0	621	72.0	454	90.0	
Marital status							0.001^a^
Single	1281	94.0	849	99.0	432	85.0	
Married	85	6.0	10	1.0	75	15.0	
Economic status							0.001^a^
Low	218	16.0	131	15.0	87	17.2	
Moderate	845	62.0	494	58.0	351	69.2	
High	303	22.0	234	27.0	69	13.6	
Parents level of Education							0.001^a^
No education/UBE	46	3.0	4	0.5	42	8.0	
High school	162	12.0	88	10.0	74	15.0	
Vocational college	167	12.0	101	12.0	66	13.0	
University	991	73.0	666	77.5	325	64.0	
Religion							0.001^a^
Adherent	513	38.0	258	30.0	255	50.2	
No religion	853	62.0	601	70.0	252	49.7	
City type							0.4^a^
Metropolis	1011	74.0	643	75.0	368	72.5	
Mega cities	339	25.0	208	24.0	131	26.0	
Missing	16	1.0	8	1.0	8	1.5	
*General health literacy*	*(N = 1307)*		*(n = 847)*		*(n = 460)*		0.0001^a^
Inadequate	784	60.0	620	73.0	164	36.0	
Sufficient	523	40.0	227	27.0	296	64.0	

^a^ Chi-squared test.

n: number of participants; UBE: Universal Basic Education.

### Association between sociodemographic factors and health status

Table 2 presents the association between sociodemographic factors and HS among university students. Good HS was positively associated with being male (*p* = 0.002), an undergraduate student (*p* = 0.008), and studying in a medical program (*p* < 0.001). It was also associated with economic status (*p* < 0.001). An inverse association was found between age and good HS (*p* = 0.003). No associations were found for marital status (*p* = 0.14), parents’ education (*p* = 0.06), religion (*p* = 0.9), or city type (*p* = 0.4). In addition, HS showed no significant variation in the proportion of international students reporting good HS based on their length of stay in Japan (*p* = 0.7) or Japanese language proficiency (*p* = 0.2). The full results are presented in Supplementary Tables 1S and 2S, respectively. Nonetheless, the highest proportion of good HS was observed among international students with a stay of less than one year.

**Table 2.** Association between sociodemographic factors and health status.

Variable	Health status (*N* = 1366) (%)				
	Good		Not Good		P a
	*n* = 934	68%	*n* = 432	32%	
Gender					0.002
Male	526	72.0	206	28.0	
Female	398	65.0	214	35.0	
others	10	45.0	12	55.0	
Age-group (years)					0.003
< 20	227	74.0	78	26.0	
20–24	489	69.0	217	31.0	
25–30	143	60.0	94	40.0	
> =31	75	64.0	43	36.0	
Level of study					0.008
Undergraduate	624	71.0	257	29.0	
Postgraduate	310	64.0	175	36.0	
Program of study					0.001
Medical	224	77.0	67	23.0	
Non-medical	710	66.0	365	34.0	
Marital status					0.1
Single	870	68.0	411	32.0	
Married	64	75.0	21	25.0	
Economic status					0.001
Low	129	59.0	89	41.0	
Moderate	573	68.0	272	32.0	
High	232	77.0	71	23.0	
Parents level of Education					0.06
No education/UBE	29	63.0	17	37.0	
High school	112	69.0	50	31.0	
Vocational college	100	60.0	67	40.0	
University	693	70.0	298	30.0	
Religion					0.9
Adherent	350	68.0	163	32.0	
No religion	584	68.0	269	32.0	
City type					0.4
Metropolis	697	69.0	314	31.0	
Mega cities	226	67.0	113	33.0	
Missing (16)					

^a^ Chi-squared test.

n: number of participants; UBE: Universal Basic Education.

### Association between nationality and health status among university students

**Figure 1.**
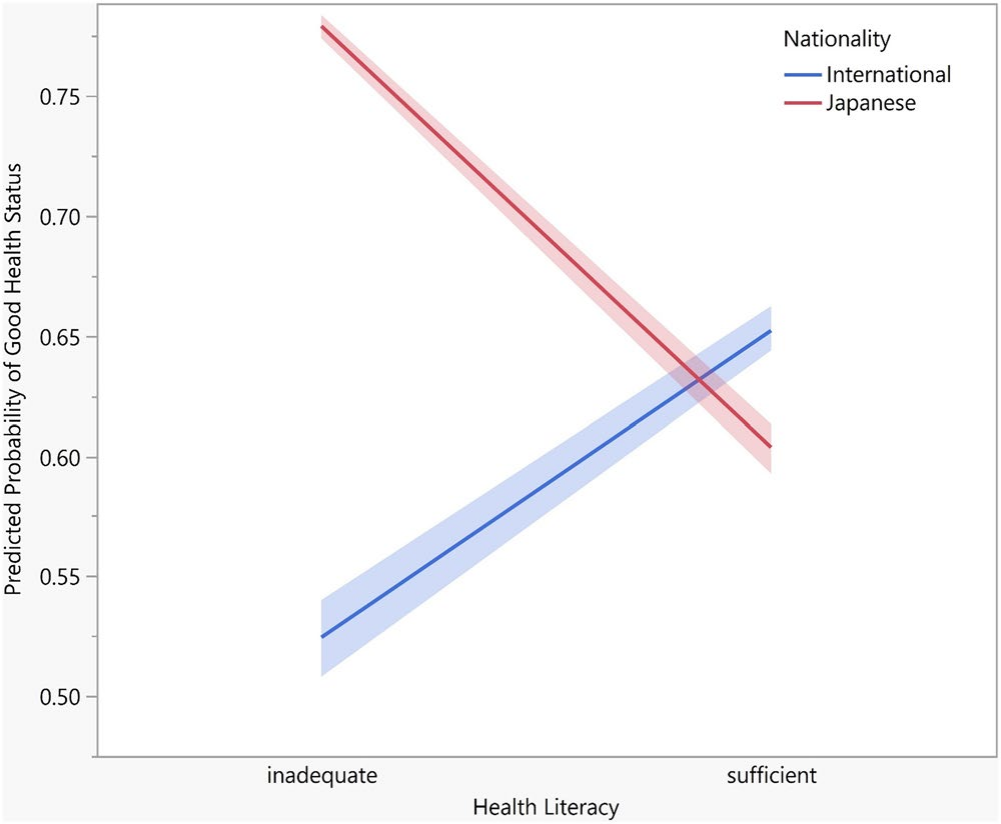
Adjusted predicted probabilities of good health status by health literacy and nationality. Predicted probabilities were derived from the fully adjusted logistic regression model including the nationality × health literacy interaction term. Shaded areas represent 95% confidence intervals.

Table 3a shows the odds ratios (ORs) and 95% confidence intervals (CIs) for the prevalence of good HS by nationality. Japanese students reported a higher proportion of good HS (73%) compared to international students (61%). The OR (95% CI) for good HS among international students, compared to Japanese students, was 0.56 (0.44 to 0.71, *p* < 0.0001). This inverse association remained after adjusting for age and sex (*p* = 0.002, Model 1). In Model 2, which additionally adjusted for sociodemographic and educational variables, international students still had a lower prevalence of good HS compared to Japanese students (OR 0.72, 95% CI 0.53 to 0.99, *p* = 0.04). When HL level was considered (Model 3), the association was no longer robust (*p* = 0.24). This pattern remained consistent in the sensitivity analyses (Supplementary Table 3a). However, the interaction between nationality and HL was observed (0.73, 95% CI: 0.64 to 0.83; *p* < 0.0001) (Table 3b; model fit shown in Supplementary Table 3b), suggesting that the association between nationality and HS varied by HL level. The marginal effects plot (Figure 1) illustrating predicted probabilities of good health status across health literacy levels by nationality, also illustrated this differing trends. Model diagnostics supported the validity of the analysis. Correlation matrix diagnostics showed no evidence of problematic multicollinearity among predictors (all correlation coefficients < 0.70). The model demonstrated modest discrimination (AUC = 0.66). Calibration assessment indicated adequate agreement between observed and predicted probabilities (Hosmer-Lemeshow *p* = 0.63). Evaluation of Cook’s distance and standardized residuals identified a small number of potentially influential observations; however, sensitivity analyses excluding these cases showed no material change in estimates.

**Table 3a.** Association between nationality and health status among university students.

	Nationality		P
	Japanese	International	
Participants, n	859	507	
Good health status, n (%)	627 (73%)	307 (61%)	
Crude	reference	0.56 (0.44–0.71)	0.0001
Model 1^a^	reference	0.64 (0.47–0.85)	0.002
Model 2^b^	reference	0.72 (0.53–0.99)	0.04
Model 3^c^	reference	0.81 (0.58–1.14)	0.2

The values are expressed as odds ratios and 95% confidence intervals. n: number of participants.

^a^ Adjusted for age and gender.

^b^ Additionally adjusted for economic status, marital status, level of study, and program of study.

^c^ Additionally adjusted for health literacy.

**Table 3b.** Interaction between nationality and health literacy in relation to health status.

	Nationality × HL		
	Japanese × Sufficient	International × Inadequate	P
Model	reference	0.73 (0.64–0.83)	0.0001

The values are expressed as adjusted odds ratios and 95% confidence intervals.

Model adjusted for age, gender, marital status, economic status, level of study, and program of study.

### Association between nationality and health status according to Health literacy level

In Table 4, the OR and 95% CIs for the prevalence of good HS among international and Japanese university students are presented according to their HL level. Among the participants with sufficient HL, 60% of Japanese students and 65% of international students reported good HS (*p* = 0.25). After adjusting for age and gender, the OR (95% CI) for good HS among international students compared to Japanese students was 1.54 (0.99 to 2.40, *p* = 0.05). When we considered sociodemographic and educational variables (Model 2), the prevalence of good HS among international students was higher than that among Japanese students (1.63 [1.03 to 2.58], *p* = 0.03). Conversely, among students with inadequate HL, 78% of Japanese students reported good HS compared to 52% of international students (*p* < 0.0001). After adjusting for all confounding factors (Model 2), this association remained robust (0.34 [0.20 to 0.58], *p* < 0.0001). Model fit statistics (−2 log likelihood, likelihood ratio chi-square statistic, AICc, BIC and pseudo-R^2^) are presented in the Supplementary Table 4a. In sensitivity analyses treating HS as an ordinal outcome (Supplementary Table 4b), the findings were consistent with those of the primary analysis. To complement the primary HL stratified analyses, we conducted additional subgroup analyses stratified by nationality to explore nationality-specific associations between HL and HS (Supplementary Table 5a and 5b). Among international students, sufficient HL was associated with better HS, whereas among Japanese students the association was in the opposite direction. Further analyses to examine the association between HL and HS across specific HL domains (healthcare, disease prevention, health promotion) and competencies (accessing, understanding, appraising, applying) (Supplementary Table 5c) showed patterns consistent with the primary composite measure, suggesting the heterogeneous role of HL across student populations.

**Table 4.** Association between nationality and health status among university students according to health literacy level.

	Nationality		P
	Japanese	International	
Sufficient HL level			
Participants, n	227	296	
Good health status, n (%)	137 (60%)	193 (65%)	
Crude	reference	1.23 (0.86–1.76)	0.2
Model 1^a^	reference	1.54 (0.99–2.40)	0.05
Model 2^b^	reference	1.63 (1.03–2.58)	0.03
Inadequate HL level			
Participants, n	620	164	
Good health status, n (%)	483 (78%)	86 (52%)	
Crude	reference	0.31 (0.21–0.44)	0.0001
Model 1^a^	reference	0.30 (0.18–0.49)	0.0001
Model 2^b^	reference	0.34 (0.20–0.58)	0.0001

The values are expressed as odds ratios and 95% confidence intervals. n: number of participants.

^a^ Adjusted for age and gender.

^b^ Additionally adjusted for economic status, marital status, level of study, and program of study.

## Discussion

The present study examined the association between nationality and HS among university students, with a particular focus on the role of HL. The findings revealed that a higher proportion of Japanese students (73%) rated their HS as good compared to international students (61%). Even after adjusting for covariates, international students showed a lower prevalence of good HS compared to their Japanese counterparts. Moreover, an interaction between HL and nationality was observed, and marginal effects plots indicate that the comparison of HS between international and Japanese university students differs among students with sufficient and inadequate HL depending on HL levels. These results suggest that although international students are generally less likely to report good HS, a lower prevalence of good HS was obtained only among international students with an inadequate HL level.

In this study, Japanese students generally reported better health than their international peers, which is inconsistent with the findings of previous studies. For instance, Skromanis and colleagues found that international students in Australia had better HS [31], whereas Umami and colleagues observed no difference between international and domestic medical students in Hungary [32]. Although our results differ from these studies, our findings are partially supported by Abe et al. [6] who confirmed poorer periodontal health (evaluated by bleeding on probing and calculus deposition) among international students compared to their domestic counterparts in Japan. One possible reason for the discrepancy between our findings and those of Skromanis et al. [31] and Umami et al. [32] may be the variations in the social structures among these countries. Japan’s relatively high living standards, safety, and stability have contributed to improved health outcomes since the establishment of universal health insurance in 1961 [46]. According to 2021 estimates from the World Health Organization, Japan leads the top seven global economies in universal healthcare coverage, with a 99% coverage rate [47]. In this study, Japanese students reported a higher economic status. Since a higher socioeconomic status is positively associated with HS [23], it is reasonable to expect Japanese students to report better HS. However, even after controlling for economic status, an association between nationality and HS was confirmed. Hence, socioeconomic status does not fully explain the association between nationality and HS. Nevertheless, these associations may partially reflect unmeasured social and psychosocial processes such as lifestyle behaviors, mental health, and social support that influence HS. Besides, the binary operationalization of nationality necessarily aggregates a heterogeneous group of international students.

Self-reported HS is a unique and valuable indicator of HS [9,10]. Given that it is unspecific, it serves as a comprehensive and inclusive measure, capturing aspects of health relevant to survival that may not be covered by other health indicators [9,11]. By integrating complex human judgments about the severity of current conditions, family history, and health trajectories over time, it also reflects the availability of resources that can mitigate health decline and influence health-related behaviors [10]. Previous studies have established that this single item self-reported HS is both valid across different ethnic groups [37] and reliable [38], correlating consistently with objective HS among the general population [39,40]. Moreover, even prospective evidence with 5-, 10-, and 27-year follow-ups shows that subjective HS outperforms objective HS in predicting mortality within unadjusted models [18]. Among adolescents and young adults, higher subjective HS has been associated with better physical and mental health, with similar amount of variance explained across racial/ethnic and immigrant generation groups [48], and has also been shown to mediate the association between subjective stress and obesity in young adults [49]. Together, these findings underscore its ability to capture holistic dimensions of health and support its appropriateness as an outcome measure in university student populations. Therefore, students’ self-assessments of their HS provide a comprehensive overview of their physical, social, and mental well-being. The findings of this study can partly be extended to immigrants, as they are likely to face similar challenges associated with migration, such as weather variations, unfamiliar food types, and socio-communication and interpersonal difficulties. This idea is supported by the findings of a previous study among elderly migrants and non-migrant populations in India [50]. However, there are limitations to this extension. International students often have access to university support systems in addition to public support services, which may contribute to a higher HS compared to the general immigrant population.

HL plays a role in modifying the association between nationality and HS among university students. This study revealed an even lower prevalence of good HS among international students with inadequate HL. In contrast, international students with sufficient HL reported a higher prevalence of good HS. Notably, the proportion of good HS among Japanese students with sufficient HL was 13 percentage points lower than that of the overall Japanese student population. HL is a multidimensional construct that extends beyond individual competencies and may also reflect prior educational trajectories, institutional familiarity, and cultural alignment with healthcare systems. This may explain why international students with sufficient HL report better HS than Japanese students with comparable HL levels. It also suggests that system-level access alone does not fully explain HS. International students with higher HL may be better positioned to selectively utilize university-based support structures and tailored services, thereby translating available resources into more favorable subjective health. Previous research has shown that higher HL levels were associated with better self-reported HS among Japanese university students [25]. Therefore, one might expect a higher proportion of good HS among Japanese students with sufficient HL than that of the overall Japanese students. The difference in findings between our study and previous research could be attributed to variations in participant characteristics, such as educational level and recruitment procedures. The consistency of findings across HL domains and competencies in sensitivity analyses suggests that the association between HL and HS by nationality was not driven by a single HL dimension but reflects a broader, multidimensional construct. However, the findings that Japanese students with sufficient HL reported lower HS than those with inadequate HS warrants careful interpretation. Higher HL may be associated with greater symptom awareness, stricter self-evaluation, or heightened health expectations, leading to lower subjective health without indicating poorer objective health. More also, HL may intersect with normative beliefs about personal responsibility for health and trust in healthcare institutions. Research on healthism suggests that greater emphasis on individual responsibility and critical appraisal of care can shape health perceptions independently of informational capacity [51,52]. This perspective may explain why higher HL does not uniformly translate into better HS across groups. Nevertheless, these findings highlight the importance of considering HL when examining HS differences between immigrant and domestic populations.

Given the observed association between HL and HS among university students from diverse nationalities, as suggested in this current study, proactive health education and promotion activities could reduce health disparities and improve health outcomes. Universities can play a pivotal role in this effort by integrating targeted HL programs and workshops into annual medical screenings and orientations, particularly for international students who may lack the necessary skills to effectively navigate the healthcare system. General health education for all students, peer-to-peer learning initiatives [22], and the adoption of the “Japanese Senpai” approach (where experienced seniors unofficially mentor junior colleagues) to propagate HL can further reinforce these efforts. Moreover, encouraging more active interactions between international and Japanese students can further enhance HL across the student body. Because HL is also shaped by long-term educational and social inequalities that may be less amenable to rapid institutional fixes, therefore, institutional efforts should be understood as complementary strategies within a broader, multi-level approach that addresses structural and educational determinants of HL.

This study has some limitations. First, the participants were recruited using a convenience sampling method and were limited to students from national universities. It is noteworthy that similar previous studies on HS and/or HL among general university student population also used web-based survey with convenient sampling for participants recruitment [25,31,32]. To enhance data accuracy and promote understanding, this current study self-administered the web-based survey. Nonetheless, this approach limits the generalizability of the findings to students with relatively high academic achievement and includes the possibility of selection bias. Second, all measurement instruments in this study were self-reported. Although both the English and Japanese versions of the HL questionnaire, as well as the single-item measure of HS, have been validated, the subjective nature of these constructs may limit measurement accuracy. Third, although this study considered several confounding factors, unmeasured and unknown confounding factors may influence the current findings, such as the specific nationality of international students, some lifestyle behaviors (e.g. diet, exercise, substance use), medical history, current physical and mental health, access to health and counselling services, levels of social interaction, social networks, support systems, perceived discrimination faced by international students, and the international experience of Japanese students. Similarly, although HL modified the association between nationality and health status, the relatively low pseudo-R^2^ values indicate that substantial variance in self-rated health remains unexplained, which is typical of subjective health outcomes and underscores that HL represents only one component within a broader set of social, behavioral, and contextual determinants; thus, the results might be overestimated or underestimated. Fourth, nationality was operationalized as a binary distinction between Japanese and international students, an approach that facilitates analysis but obscures substantial heterogeneity within the international student population. Although supplementary analyses showed no association between length of stay or Japanese language proficiency and HS, international students should not be interpreted as a homogeneous group. The observed associations likely reflect broader structural and contextual differences related to migrant status rather than individual-level characteristics, and future studies should explore within-group variation among international students. Finally, the cross-sectional design of the study limits our ability to infer causality. Although nationality is a constant attribute, the association between HL and HS may be bidirectional, and reverse causality cannot be ruled out. Hence, longitudinal studies would be more beneficial for exploring the association between nationality and HS and the role of HL in this association.

## Conclusion

Our study showed that international students reported a lower prevalence of good HS compared to their Japanese counterparts. Moreover, an interaction between HL and nationality was observed, indicating that the association between nationality and HS differs among students depending on HL levels. Although international students with inadequate HL had a lower prevalence of good HS, those with sufficient HL had a higher prevalence of reporting good HS.

## Supplementary Material

Supplementary Tables.docx

## Data Availability

The data that support the findings of this research are available from the corresponding author, A.A, upon reasonable request.
