# Supplementary material for: The role of health literacy in the association between nationality and health status among university students: a cross-sectional study
Source: Ann Med. 2026 Mar 26;58(1):2643976. doi: 10.1080/07853890.2026.2643976 (PMC13023008; doi:10.1080/07853890.2026.2643976)
Supplement: Supplementary Tables.docx [file IANN_A_2643976_SM5798.docx]

**Supplementary Tables**

Supplementary Table 1S: **Association between length of stay and health status among international students**

| Variable | | Health Status | | | | |
| --- | --- | --- | --- | --- | --- | --- |
|  |  | Good | | Not Good | | P **^a^** |
|  |  | n = 303 | 60% | n = 198 | 40% |  |
| **Length of stay in Japan**  **(N= 501)** | < 1yr | 109 | 63.0 | 64 | 37.0 | 0.7 |
|  | 1-2yrs | 96 | 61.0 | 61 | 39.0 |  |
|  | 3-4yrs | 59 | 56.0 | 46 | 44.0 |  |
|  | >=5yrs | 39 | 59.0 | 27 | 41.0 |  |

**^a^** Chi-squared test.

n: number of participants

Supplementary Table 2S: **Association between Japanese language proficiency and health status among international students**

| Variable | | Health Status | | | | |
| --- | --- | --- | --- | --- | --- | --- |
|  |  | Good | | Not Good | | P **^a^** |
|  |  | n = 307 | 61% | n = 200 | 39% |  |
| **Japanese Language Proficiency**  **(N= 507)** | Cannot speak | 31 | 55.0 | 25 | 45.0 | 0.2 |
|  | Can speak a little | 116 | 68.0 | 55 | 32.0 |  |
|  | Able to communicate about daily life | 111 | 57.0 | 83 | 43.0 |  |
|  | Able to report and understand information using medical terms | 15 | 56.0 | 12 | 44.0 |  |
|  | Speaks fluently | 34 | 58.0 | 25 | 42.0 |  |

**^a^** Chi-squared test.

n: number of participants

Supplementary Table 3a **Association between nationality and health status among university students**

|  | Nationality (N = 1307) | | | P |
| --- | --- | --- | --- | --- |
|  | Japanese | International | |  |
|  |  | β (SE) | 95 % CI |  |
| Crude | reference | –0.13 (0.02) | –0.17 – (–0.08) | 0.0001 |
| Model 1^a^ | reference | –0.08 (0.02) | –0.13 – (–0.02) | 0.005 |
| Model 2^b^ | reference | –0.02 (0.03) | –0.09 – (–0.03) | 0.3 |
| *R-squared (Adjusted R-squared) = ^crude^ 0.02 (0.02), ^a^0.06 (0.06), ^b^0.08 (0.07)* | | | | |

CI: confidence interval, β: estimate, SE: standard error. N: number of participants.

**^a^** Adjusted for age and gender, economic status, marital status, level of study, and program of study.

**^b^** Additionally adjusted for health literacy

Supplementary Table 3b. **The model fit statistics for adjusted regression model examining the interaction between nationality and health literacy in relation to health status**

|  | −2LL | Wald χ² | AICc | BIC | β (SE) |
| --- | --- | --- | --- | --- | --- |
| Nationality × HL | *1530.50* | 22.27 | *1558.82* | *1630.96* | –0.31 (0.06) |

Nationality × HL: International × Inadequate HL (reference: Japanese × Sufficient HL)

Model adjusted for age, gender, marital status, economic status, level of study, and program of study. −2LL: 2 log likelihood, Wald χ²: Wald chi-square statistic AIC and BIC: Akaike and Bayesian information criteria, β: estimate, SE: standard error, HL: health literacy

Supplementary Table 4a. **Model fit statistics for fully adjusted logistic regression models examining the association between nationality and health status, according to health literacy level**

|  | −2LL | LR χ² (df = 11) | AICc | BIC | Cox & Snell R² |
| --- | --- | --- | --- | --- | --- |
| Sufficient HL level | 660.72 | 28.00 | 685.34 | 735.84 | 0.04 |
| Inadequate HL level | 859.69 | 61.39 | 884.10 | 939.66 | 0.06 |

Nationality: International (reference: Japanese)

Model adjusted for age, gender, economic status, marital status, level of study, and program of study. −2LL: −2 log likelihood, LR χ²: likelihood ratio chi-square statistic, AIC and BIC: Akaike and Bayesian information criteria, Cox & Snell R² represents the pseudo-R², HL: health literacy

Supplementary Table 4b: **Association between Nationality and health status among university students according to health literacy level**

|  | Nationality | | | P |
| --- | --- | --- | --- | --- |
|  | Japanese | International | |  |
| Sufficient HL level (N= 460) |  | β (SE) | 95 % CI |  |
| Crude | reference | 0.03 (0.03) | –0.03 – 0.09 | 0.3 |
| Model 1^a^ | reference | 0.05 (0.04) | –0.02 – 0.13 | 0.1 |
| Model 2^b^ | reference | 0.07 (0.04) | –0.01 – 0.15 | 0.08 |
| *R-squared (Adjusted R-squared) = ^crude^ 0.00 (–0.00), ^a^0.02 (0.00), ^b^0.07 (0.05)* | | | | |
| Inadequate HL level (N= 874) |  |  |  |  |
| Crude | reference | –0.24 (0.03) | –0.31 – (–0.17) | 0.0001 |
| Model 1^a^ | reference | –0.22 (0.04) | –0.31 – (–0.13) | 0.0001 |
| Model 2^b^ | reference | –0.19 (0.04) | –0.29 – (–0.10) | 0.0001 |
| *R-squared (Adjusted R-squared) = ^crude^ 0.05 (0.05), ^a^0.06 (0.05), ^b^0.08 (0.07)* | | | | |

CI: confidence interval, β: estimate, SE: standard error. N: number of participants.

**^a^** Adjusted for age and gender.

**^b^** Additionally adjusted for economic status, marital status, level of study, and program of study.

Supplementary Table 5a: **Association between health literacy and health status among university students by nationality.**

|  | Health Literacy Level | | P |
| --- | --- | --- | --- |
|  | Inadequate | Sufficient |  |
| International students |  |  |  |
| Participants, n | 164 | 296 |  |
| Good health status, n (%) | 86 (52%) | 193 (65%) |  |
| Crude | reference | 1.69 (1.15 – 2.50) | 0.007 |
| Model 1^a^ | reference | 1.65 (1.10 – 2.46) | 0.01 |
| Model 2^b^ | reference | 1.59 (1.06 – 2.41) | 0.02 |
| *fully adjusted* ^b^*Model fit: −2LL = 582.55; LR χ² (df = 11) = 34.11; AICc = 607.24; BIC = 656.12; Cox & Snell R² = 0.05, N = 460* | | | |
| Japanese students |  |  |  |
| Participants, n | 620 | 227 |  |
| Good health status, n (%) | 483 (78%) | 137 (60%) |  |
| Crude | reference | 0.43 (0.31 – 0.59) | 0.0001 |
| Model 1^a^ | reference | 0.43 (0.31 – 0.61) | 0.0001 |
| Model 2^b^ | reference | 0.45 (0.32 – 0.63) | 0.0001 |
| *fully adjusted* ^b^*Model fit: −2LL = 937.88; LR χ² (df = 11) = 46.79; AICc = 962.25; BIC = 1018.78; Cox & Snell R² = 0.04; N = 847* | | | |

The values are expressed as odds ratios and 95% confidence intervals. n: number of participants.

−2LL: −2 log likelihood, LR χ²: likelihood ratio chi-square statistic, AIC and BIC: Akaike and Bayesian information criteria, Cox & Snell R² represents the pseudo-R².

**^a^** Adjusted for age and gender.

**^b^** Additionally adjusted for economic status, marital status, level of study, and program of study.

**Table 5b Association between health literacy and health status among university students by Nationality**

| Nationality | β (SE) | 95 % CI | P |
| --- | --- | --- | --- |
| International (N= 460) |  |  |  |
| Crude | 0.01 (0.004) | 0.005 – 0.023 | 0.001 |
| Model 1^a^ | 0.01 (0.004) | 0.002 – 0.020 | 0.01 |
| *R-squared (Adjusted R-squared) = ^crude^ 0.02 (0.01), ^a^0.10 (0.08)* | | | |
| Japanese (N= 874) |  |  |  |
| Crude | –0.03 (0.003) | –0.039 – (–0.025) | 0.0001 |
| Model 1^a^ | –0.03 (0.003) | –0.038 – (–0.022) | 0.0001 |
| *R-squared (Adjusted R-squared) = ^crude^ 0.07 (0.07), ^a^0.10 (0.09)* | | | |

CI: confidence interval, β: estimate, SE: standard error. N: number of participants.

**^a^** Adjusted for age and gender, economic status, marital status, level of study, and program of study.

**Table 5c Association between health literacy domains and competence and health status among university students by Nationality**

| Nationality | N | Model | OR (95% CI) | P |
| --- | --- | --- | --- | --- |
| International |  |  |  |  |
| Health literacy domain |  |  |  |  |
| *Healthcare (n=457)* | 457 | Crude | 1.03 (1.00 – 1.05) | 0.009 |
|  |  | Model 1^a^ | 1.03 (1.00 – 1.05) | 0.02 |
| *Disease prevention* | 462 | Crude | 1.03 (1.01 – 1.05) | 0.003 |
|  |  | Model 1^a^ | 1.02 (1.00 – 1.05) | 0.02 |
| *Health promotion* | 473 | Crude | 1.02 (1.00 – 1.04) | 0.01 |
|  |  | Model 1^a^ | 1.02 (0.99 – 1.04) | 0.06 |
| Health literacy competence |  |  |  |  |
| *Accessing* | 443 | Crude | 1.03 (1.00 – 1.05) | 0.009 |
|  |  | Model 1^a^ | 1.02 (1.00 – 1.04) | 0.04 |
| *Understanding* | 481 | Crude | 1.02 (1.00 – 1.04) | 0.008 |
|  |  | Model 1^a^ | 1.02 (1.00 – 1.04) | 0.02 |
| *Appraising* | 444 | Crude | 1.01 (0.99 – 1.04) | 0.1 |
|  |  | Model 1^a^ | 1.01 (0.98 – 1.03) | 0.3 |
| *Applying* | 464 | Crude | 1.02 (1.00 – 1.04) | 0.01 |
|  |  | Model 1^a^ | 1.02 (1.00 – 1.04) | 0.06 |
| Japanese |  |  |  |  |
| Health literacy domain |  |  |  |  |
| *Healthcare* | 843 | Crude | 0.95 (0.93 – 0.97) | 0.0001 |
|  |  | Model 1^a^ | 0.95 (0.93 – 0.97) | 0.0001 |
| *Disease prevention* | 848 | Crude | 0.94 (0.92 – 0.96) | 0.0001 |
|  |  | Model 1^a^ | 0.95 (0.93 – 0.96) | 0.0001 |
| *Health promotion* | 848 | Crude | 0.94 (0.92 – 0.95) | 0.0001 |
|  |  | Model 1^a^ | 0.94 (0.92 – 0.96) | 0.0001 |
| Health literacy competence |  |  |  |  |
| *Accessing* | 830 | Crude | 0.94 (0.92 – 0.96) | 0.0001 |
|  |  | Model 1^a^ | 0.94 (0.92 – 0.96) | 0.0001 |
| *Understanding* | 852 | Crude | 0.95 (0.93 – 0.97) | 0.0001 |
|  |  | Model 1^a^ | 0.95 (0.93 – 0.97) | 0.0001 |
| *Appraising* | 841 | Crude | 0.96 (0.95 – 0.98) | 0.0002 |
|  |  | Model 1^a^ | 0.97 (0.95 – 0.98) | 0.001 |
| *Applying* | 850 | Crude | 0.93 (0.91 – 0.95) | 0.0001 |
|  |  | Model 1^a^ | 0.93 (0.91 – 0.95) | 0.0001 |

The values are expressed as odds ratios and 95% confidence intervals. n: number of participants.

Odds ratios represent the change in odds of reporting good (vs poor) health status per one unit increase in each health literacy domain and competence score

**^a^** Adjusted for age and gender, economic status, marital status, level of study, and program of study.
